# A Physician’s Counsels to Woman in Health and Disease

**Published:** 1871-08

**Authors:** 


A Physician's Counsels to Woman in Health and Disease, by Walter C. Taylor, A.M., M.D., author of Gynæcological Notes, etc. Springfield: W. J. Holland & Co., publishers.

